# Correction: Body mass index and severity/fatality from coronavirus disease 2019: A nationwide epidemiological study in Korea

**DOI:** 10.1371/journal.pone.0301962

**Published:** 2024-04-04

**Authors:** In Sook Kang, Kyoung Ae Kong

In Fig 1, there are typographical errors in the BMI values. The ranges 2.3–24.9 and 2.5–29.9 should be 23.0–24.9 and 25.0–29.9, respectively. Please see the correct Fig 1 here.

**Fig 1:**
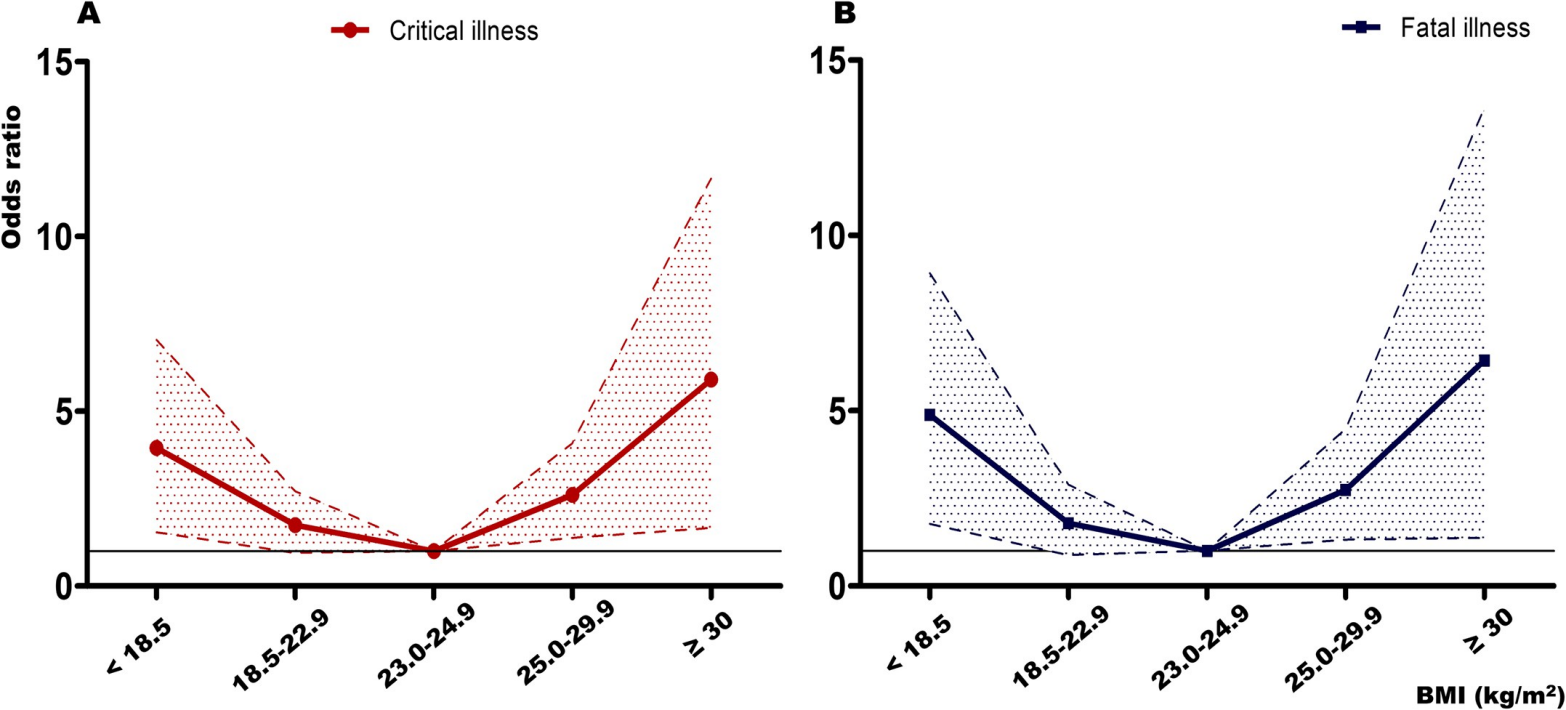
Odds ratios for critical and fatal illness according to body mass index.

Odds ratios were adjusted for age, gender, and five comorbidities (diabetes mellitus, hypertension, chronic kidney disease, cancer, and dementia).
