# Supplementary material for: Body mass index and severity/fatality from coronavirus disease 2019: A nationwide epidemiological study in Korea
Source: PLoS One. 2021 Jun 22;16(6):e0253640. doi: 10.1371/journal.pone.0253640 (PMC8219144; doi:10.1371/journal.pone.0253640)
Supplement: S1 Table — (DOCX) [file pone.0253640.s001.docx]

**S1 Table**. **Baseline characteristics of the study population according to disease severity**

|  | **Total** | **Severity** | |  |
| --- | --- | --- | --- | --- |
|  |  | **Non-fatal illness** | **Fatal illness** | ***P*** |
| **N** | **4141** | **4015** | **126** |  |
| **Age (years)** |  |  |  | <0.0001 |
| 20-29 | 942 (22.7) | 942 (23.5) | 0 (0) |  |
| 30-39 | 469 (11.3) | 469 (11.7) | 0 (0) |  |
| 40-49 | 595 (14.4) | 594 (14.8) | 1 (0.8) |  |
| 50-59 | 873 (21.1) | 865 (21.5) | 8 (6.3) |  |
| 60-69 | 674 (16.3) | 656 (16.3) | 18 (14.3) |  |
| 70-79 | 378 ( 9.1) | 336 (8.4) | 42 (33.3) |  |
| ≥ 80 | 210 (5.1) | 153 (3.8) | 57 (45.2) |  |
| **Male** | 1726 (41.7) | 1654 (41.2) | 72 (57.1) | 0.0004 |
| **Dyspnea** |  |  |  | <0.0001 |
| Yes | 492 (11.9) | 425 (10.6) | 67 (53.2) |  |
| No | 3648 (88.1) | 3589 (89.4) | 59 (46.8) |  |
| **SBP* (mmHg)** |  |  |  | <0.0001 |
| <120 | 964 (23.3) | 935 (23.3) | 29 (23.2) |  |
| 120-129 | 880 (21.3) | 861 (21.5) | 19 (15.2) |  |
| 130-139 | 840 (20.3) | 826 (20.6) | 14 (11.2) |  |
| 140-159 | 1078 (26.0) | 1039 (25.9) | 39 (31.2) |  |
| ≥ 160 | 374 (9.0) | 350 (8.7) | 24 (19.2) |  |
| **Diabetes mellitus** |  |  |  | <0.0001 |
| Yes | 501 (12.1) | 445 (11.1) | 56 (44.4) |  |
| No | 3640 (87.9) | 3570 (88.9) | 70 (55.6) |  |
| **Hypertension** |  |  |  | <0.0001 |
| Yes | 862 (20.8) | 785 (19.6) | 77 (61.1) |  |
| No | 3279 (79.2) | 3230 (80.4) | 49 (38.9) |  |
| **Heart failure** |  |  |  | <0.0001 |
| Yes | 40 (1.0) | 30 (0.7) | 10 (7.9) |  |
| No | 4101 (99.0) | 3985 (99.3) | 116 (92.1) |  |
| **CHD†** |  |  |  | <0.0001 |
| Yes | 134 (3.2) | 119 (3.0) | 15 (11.9) |  |
| No | 3991 (96.4) | 3880 (84.2) | 111 (88.1) |  |
| **Asthma** |  |  |  | 0.0028 |
| Yes | 98 (2.4) | 90 (2.2) | 8 (6.3) |  |
| No | 4043 (97.6) | 3925 (97.8) | 118 (93.7) |  |
| **COPD** |  |  |  | 0.0007 |
| Yes | 29 (0.7) | 25 (0.6) | 4 (3.2) |  |
| No | 4112 (99.3) | 3990 (99.4) | 122 (96.8) |  |
| **CKD** |  |  |  | <0.0001 |
| Yes | 44 (1.1) | 34 (0.8) | 10 (7.9) |  |
| No | 4097 (98.9) | 3981 (99.2) | 116 (92.1) |  |
| **Malignancy** |  |  |  | <0.0001 |
| Yes | 107 (2.6) | 94 (2.3) | 13 (10.3) |  |
| No | 4034 (97.4) | 3921 (97.7) | 113 (89.7) |  |
| **Dementia**‡ |  |  |  | <0.0001 |
| Yes | 121 (2.9) | 86 (2.1) | 35 (27.8) |  |
| No | 3706 (89.5) | 3084 (76.8) | 91 (72.2) |  |
| **Disease severity** |  |  |  | <0.0001 |
| No limitation | 3289 (79.4) | 3289 (81.9) | 0 (0) |  |
| Limitation but no oxygen | 276 (6.7) | 276 (6.9) | 0 (0) |  |
| Nasal oxygen | 365 (8.8) | 365 (9.1) | 0 (0) |  |
| Mask oxygen | 30 (0.7) | 30 (0.7) | 0 (0) |  |
| Noninvasive oxygen | 29 (0.7) | 29 (0.7) | 0 (0) |  |
| Invasive ventilation | 16 (0.4) | 16 (0.4) | 0 (0) |  |
| Multi-organ failure | 10 (0.2) | 10 (0.2) | 0 (0) |  |
| Death | 126 (3.0) | 0 (0) | 126 (100) |  |

SBP, systolic blood pressure; CHD, chronic heart disease; COPD, chronic obstructive lung disease; CKD, chronic kidney disease

Missing data; * n=5, **†** n= 16, ‡ n=314
