# Supplementary figures and images for: Body mass index and severity/fatality from coronavirus disease 2019: A nationwide epidemiological study in Korea
Source: PLoS One. 2021 Jun 22;16(6):e0253640. doi: 10.1371/journal.pone.0253640 (PMC8219144; doi:10.1371/journal.pone.0253640)

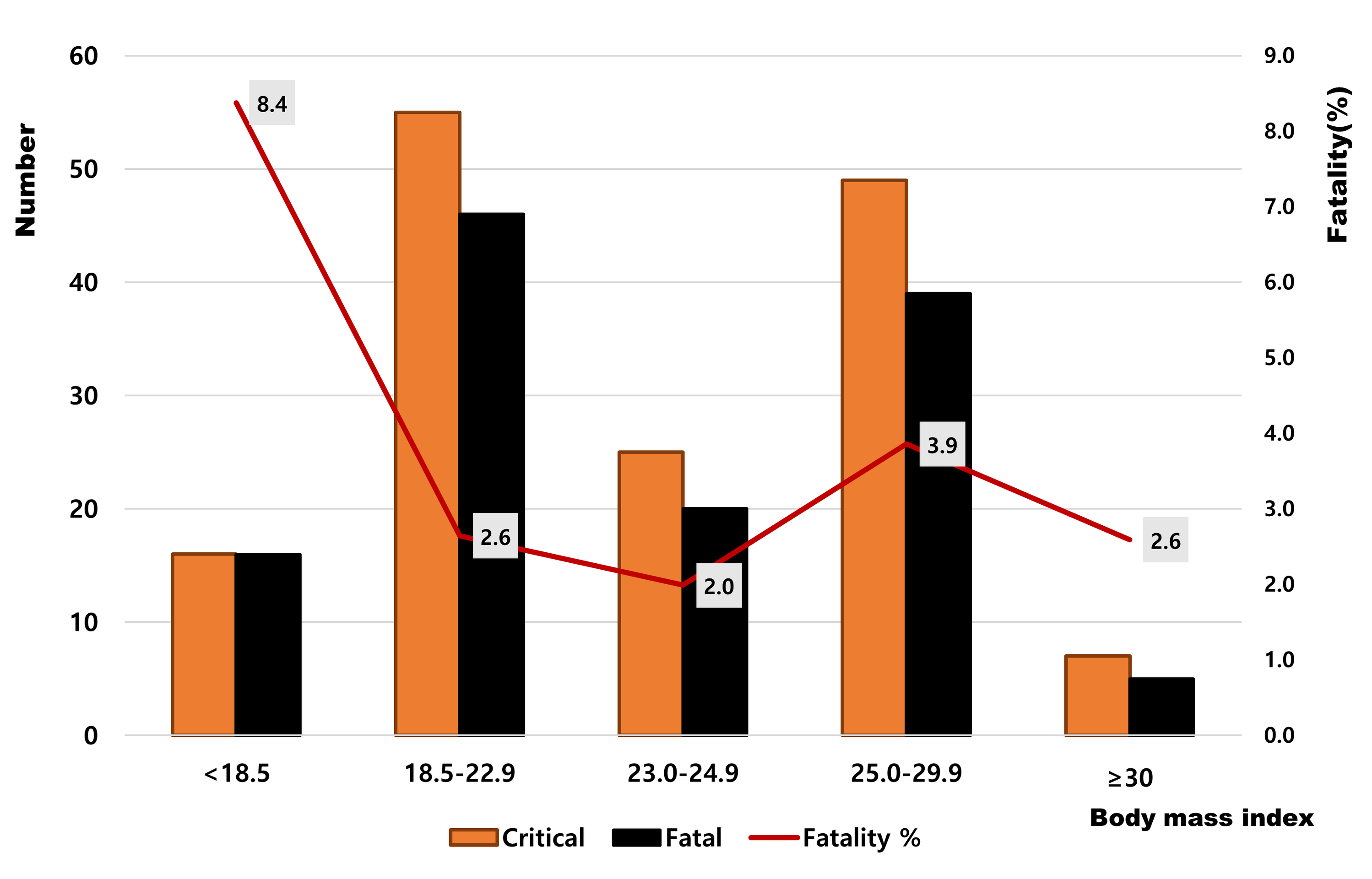

Supplement: S1 Fig — The left y-axis presents bar graphs showing the number of critical and fatal illnesses, and the right y-axis presents as a line graph showing fatality. (TIF) [file pone.0253640.s002.tif]
