# Supplementary material for: Body mass index and severity/fatality from coronavirus disease 2019: A nationwide epidemiological study in Korea
Source: PLoS One. 2021 Jun 22;16(6):e0253640. doi: 10.1371/journal.pone.0253640 (PMC8219144; doi:10.1371/journal.pone.0253640)
Supplement: Attachment — Submitted filename: 1. response to reviewers_ver 4.2.docx [file pone.0253640.s003.docx]

**In reply to Reviewer 1**

**We deeply appreciate the Reviewer for his/her time and input. We have carefully considered the reviewer’s comments and have addressed them point-by-point.**

**Comments to the Author**

**1. Reviewer commented:**

**This study investigated the relationship between BMI and COVID-19 fatal illness in a Korean sample. While this study is of quality, it can be improved in the following parts.**

**1. The introduction cited some studies about the relationship between BMI and severe COVID-19 illness. However, these studies are observational studies only and cannot provide high level evidence about the causation. There are several Mendelian randomisation studies on this relationship, such as Ponsford et al Circulation 2020 & Li et al BMC Medical Genomics 2021. These studies should be cited to give a more comprehensive review.**

**Authors’ response:** We appreciated the Reviewer’s comments and good information. We added some sentence at Introduction as below.

**Introduction, page 3**

From a study in China, obese men (BMI ≥ 28 kg/m^2^) were associated with increased risk of severe COVID-19 than normal weight men (BMI < 24 kg/m^2^) with an odds ratio of 5.66 (95% CI; 1.8-17.75) [9]. Recent studies using Mendelian randomization showed a higher genetically proxied BMI-related increasing risk of severe respiratory COVID-19 and COVID-19 hospitalization [10, 11]

**2. BMI is a continuous variable, but this study interestingly analysed it as a categorical variable. Transforming continuous variable to categorical would lose information. The study should provide the results for BMI as a continuous variable as well, and more importantly, to investigate if the U-shape still exists after using continuous BMI.**

**Authors’ response:** Thank you for good comment. However, we did not have BMI as continuous value. Given data from the Korean Centers for Disease Control and Prevention Agency was already categorized.

**3. The study found a U-shape, which is interesting, especially for the underweight group. Table 1 shows that young people have the highest proportion of underweight people; however, young people have a low risk of fatal illness. So the results are a ‘paradox’. It’s good to report and check the distribution of continuous BMI by fatal illness status.**

**Authors’ response:** Unfortunately, we did not have BMI as continuous value. The Korea Disease Control and Prevention Agency (KDCA) provided BMI values as categorized form and did not give us the actual values. It may be due to concerns about personal information. We added a sentence at discussion as limitation.

**Discussion, page 18**

Furthermore, we could not check the distribution of continuous BMI according to fatal illness status. The KCDA provided BMI data in a categorized form.

**4. The results for the underweight group could be driven by the small size of this group (so the confidence interval is very wide). Conclusion based on this small number could be misleading. The authors should acknowledge this limitation and be cautious about their conclusion.**

**Authors’ response:** We agreed your comments and added at Discussion as limitation.

**Discussion, page 18**

Although the fatality of BMI and COVID-19 was evaluated by adjusting for sex, age, and comorbid conditions, there was an issue in subgroup analysis because the sample was insufficient in terms of specific diseases. Furthermore, detailed information about comorbidities was not provided. The number of patients with BMI < 18.5, or ≥ 30 kg/m^2^, was small; 191 (4.6%) or 193 (4.7%), respectively. Therefore, there is a chance of being statistically underpowered or having an increased margin of error.

**5. The authors interestingly used overweight group, rather than normal weight group, as the reference in their logistic regression. They justified this by stating that the overweight group had the lowest fatality rate. However, this is not a good justification, and the resulted ORs have little practical usage (because they are not compared with normal weight). Weight group should be the reference out of nature and intuition. The study should use the normal weight group as reference and report new results.**

**Authors’ response:** Thank you for the good comment. Regarding the opinion of the reviewer, we conducted statistical analysis based on the Asian-pacific cutoff value and the global standard (Table A). The results still showed U-shaped relationship. The authors discussed a lot about this issue. The overweight group (23.0-24.9 kg/m^2^) of this study is according to the Asian-pacific cutoff value redefined in 2000 and which corresponds to the normal weight according to the global WHO standard. There is still a debate over the Asian-pacific cutoff value. In 2004, WHO expert consultation recommended that the use of global standards is appropriate because the BMI standards for obesity do not differ greatly by race, and it is not appropriate to stipulate different standards for the Asia-Pacific Region due to small differences. Therefore, it is the opinion of the authors not to change the reference group. However, in order to avoid confusion of global readers, the name of groups such as normal or overweight have been deleted and displayed as numerical values.

Further, the point of this study is to look at the criticality or fatality of covid-19 according to BMI, and it is not to verify the definition of the BMI cutoff value of the Korean or Asian population by comparing it with the WHO standard. However, this is considered to be a very important issue, and it is worth discussing, and the content was added at the discussion as below.

**Discussion, page 13-14**

Obesity, defined using BMI, has different cutoff values between the global and Asia-Pacific standards. The WHO announced the Asia-Pacific perspective: redefining obesity and its treatment’ in 2000 on the evidence that the Asian population has an increased risk of diseases at BMI > 23 kg/m^2^ [21]. The Asia-Pacific BMI was categorized as followings; underweight (< 18.5 kg/m^2^), normal-weight (18.5-22.9 kg/m^2^), overweight (23.0-24.9 kg/m^2^), obesity-1 (25.0-29.9 kg/m^2^), and obesity-2 (≥ 30 kg/m^2^). Proposed mechanisms of this inter-population discrepancy of defined obesity by BMI include differences in percentage and distribution of body fat across populations, the interplay of genetic susceptibility, and environmental factors related to diet and sedentary lifestyle [19, 20].

However, the debate on whether racial differences exist in defining obesity based on BMI is still ongoing. Despite the differences in BMI cutoff points for obesity in these populations, a meta-analysis of four continents in 239 prospective studies showed that BMI and all-cause mortality were broadly consistent across the four continents [3]. In 2004, WHO expert consultation recommended that the use of global standards is appropriate because the BMI standards for obesity do not differ greatly by race, and it is not appropriate to stipulate different standards for the Asia-Pacific region due to small differences [19]. In a study comparing the BMI cutoff value of the South Koreans with the global standard in 2015, the cutoff point of Korean BMI was 24.2 kg/m^2^ (sensitivity 78%, specificity 71%, as the result of receiver operating characteristic cure, based on the body fat percentage) and it was only 1.3 kg/m^2^ lower than the global standard [25]. The result supports the recommendation of the WHO expert consultation in 2004. Nonetheless, the Korean government continues to follow the 2000 Asian-Pacific cut-off values. Hence, to avoid confusion, we did not express obesity or normal for the BMI group, but only used numeric values in this study.

**Table A. Multivariable regression according to the different BMI reference**

|  | **Critical illness (original data)** | **Critical illness (Asia-Pacific)** | **Critical illness (Global )** |
| --- | --- | --- | --- |
|  | **Odds ratio (95% CI)** | **Odds ratio (95% CI)** | **Odds ratio (95% CI)** |
| **Age** | 2.64 (2.20-3.15) | 2.64 (2.20-3.15) | 2.64 (2.21-3.16) |
| **Male (vs. female)** | 2.85 (1.94-4.19) | 2.85 (1.94-4.19) | 2.79 (1.90-4.09) |
| **BMI (kg/m^2^)** |  |  |  |
| <18.5 | 3.29 (1.54-7.04) | 2.07 (1.05-4.09) | 2.41 (1.24-4.68) |
| 18.5-22.9 | 1.59 (0.94-2.71) | 1 (Reference) | 1 (Reference) |
| 23-24.9 | 1 (Reference) | 0.63 (0.37-1.07) |  |
| 25.0-29.9 | 2.37 (1.38-4.07) | 1.49 (0.94-2.36) | 1.79 (1.81-2.71) |
| ≥30 | 4.40 (1.66-11.66) | 2.76 (1.09-7.00) | 3.31 (1.33-8.22) |
| **Diabetes mellitus** | 1.95 (1.32-2.86) | 1.95 (1.32-2.86) | 1.90 (1.30-2.80) |
| **Hypertension** | 1.64 (1.11-2.43) | 1.64 (1.11-2.43) | 1.61 (1.09-2.38) |
| **Chronic kidney disease** | 3.12 (1.36-7.19) | 3.12 (1.36-7.19) | 3.17 (1.38-7.82) |
| **Cancer** | 2.28 (1.12-4.62) | 2.28 (1.12-4.62) | 3.17 (1.38-7.28) |
| **Dementia** | 2.13 (1.25-3.63) | 2.13 (1.25-3.63) | 2.22 (1.30-3.78) |

**In reply to Reviewer 2**

**We deeply appreciate the Reviewer for his/her time and input. We have carefully considered the reviewer’s comments and have addressed them point-by-point.**

**Comments to the Author**

**Please see below for my general comments on the Manuscript: Body mass index and fatality from coronavirus disease 2019: A nationwide epidemiological study in Korea. I have provided detailed feedback and questions regarding the methods, results, and interpretation of this analysis - both general comments/questions as well as line-specific. If my suggested analytical updates are made and thorough copy editing is completed, I believe this could add to the body of evidence around BMI and COVID-19 severity and fatality.

General comments/questions:
1. The manuscript has poor English grammar, and requires copy-editing for greater clarity.**

**Authors’ response:** We had already done English Editing service. However, we re-performed English Editing service, according to your comment.

**2. The authors should doing a sensitivity analysis with the global BMI cut-points for underweight, normal weight, overweight and obesity. It would be interesting to see if there is a difference, given these Asia-Pacific population cut-points are relatively new and less widely used (which they discuss in the discussion but don't analyze)**

**Authors’ response:** If we had reference standards such as body fat percentage, we could able to perform a sensitivity analysis with the global BMI and Asia-Pacific population cut-points. We did not have such an information. Further, the point of this study is to look at the criticality or fatality of covid-19 according to BMI, and it is not to verify the definition of the BMI cutoff value of the Korean or Asian population by comparing it with the WHO standard.

However, we agreed this issue was very important. There is a study showed the sensitivity and specificity of Western and Asia-Pacific BMI cut-point for Korean population using body fat percentage and we added the content at the discussion as below.

**Discussion, page 13-14**

Obesity, defined using BMI, has different cutoff values between the global and Asia-Pacific standards. The WHO announced the Asia-Pacific perspective: redefining obesity and its treatment’ in 2000 on the evidence that the Asian population has an increased risk of diseases at BMI > 23 kg/m^2^ [21]. The Asia-Pacific BMI was categorized as followings; underweight (< 18.5 kg/m^2^), normal-weight (18.5-22.9 kg/m^2^), overweight (23.0-24.9 kg/m^2^), obesity-1 (25.0-29.9 kg/m^2^), and obesity-2 (≥ 30 kg/m^2^). Proposed mechanisms of this inter-population discrepancy of defined obesity by BMI include differences in percentage and distribution of body fat across populations, the interplay of genetic susceptibility, and environmental factors related to diet and sedentary lifestyle [19, 20].

However, the debate on whether racial differences exist in defining obesity based on BMI is still ongoing. Despite the differences in BMI cutoff points for obesity in these populations, a meta-analysis of four continents in 239 prospective studies showed that BMI and all-cause mortality were broadly consistent across the four continents [3]. In 2004, WHO expert consultation recommended that the use of global standards is appropriate because the BMI standards for obesity do not differ greatly by race, and it is not appropriate to stipulate different standards for the Asia-Pacific region due to small differences [19]. In a study comparing the BMI cutoff value of the South Koreans with the global standard in 2015, the cutoff point of Korean BMI was 24.2 kg/m^2^ (sensitivity 78%, specificity 71%, as the result of receiver operating characteristic cure, based on the body fat percentage) and it was only 1.3 kg/m^2^ lower than the global standard [25]. The result supports the recommendation of the WHO expert consultation in 2004. Nonetheless, the Korean government continues to follow the 2000 Asian-Pacific cut-off values. Hence, to avoid confusion, we did not express obesity or normal for the BMI group, but only used numeric values in this study.

**3. The authors should provide definitions of each of the co-morbid conditions (self-report, taking medications), particularly for cancer – which cancers did they include?
Authors’ response:** We did not have detailed information regarding the co-morbid condition. This is the weak point of the nation-wide data. The co-morbid condition was collected through history taking by medical personnel. There was no information regarding medications. Also, we didn’t have detailed information such as cancer types but cured cancers were excluded.

**Discussion, page 18**

Further, detailed information about comorbidities were not provided.

**Statistical Analysis: general comments/questions:
1.How did the authors account for the correlation between the various risk factors as well as potential mediating affects i.e. BMI as a mediator of diabetes)?**

**Authors’ response:** We regarded diabetes and other comorbid conditions as confounders rather than mediators (that is, the intermediate variable in the causal pathway between them) to the relationship of BMI and critical disease/mortality among COVID-19 infected persons. Diabetes and other comorbidities are all chronic diseases and conditions. If we could observe for a long time, whether BMI cause diabetes or other comorbid conditions. Then, we could assess whether the diabetes or other comorbid condition might be another risk factor for the severe COVID-19.

Regarding our topic, it is hard to postulate that higher/lower BMI caused diabetes or other comorbid condition and then the comorbidities caused the criticality/fatality of COVID-19 as mediators. Hence, we performed multivariable logistic regression to analyze the independent risk for critical and fatal illness after adjusting age, sex, BMI, and five comorbid diseases: diabetes mellitus, hypertension, chronic kidney disease, cancer (excluding cured cases), and dementia as already described at statistical analysis of introduction.

As you know, “the strength of multivariable analysis is its ability to determine how multiple independent variables, which are related to one another, are related to an outcome. We would not need multivariable analysis to determine the independent effect of the variable of interest on outcome if it weren’t for the fact that independent variables were all related to each other and the outcome of interest. Multivariable analysis helps us to separate the effects of these different variables on outcomes … .” (from Multivariable analysis, 3rd ed. Mitchell H. Katz).

Further, the variables in our model were not so severely correlated that they should not be included in the model (Table A).

**Table A. Spearman’s correlation**

|  | Age | Sex (M=1) | BMI | DM | HTN | CKD | Cancer | Dementia |
| --- | --- | --- | --- | --- | --- | --- | --- | --- |
| Age | 1 | -0.08* | 0.03 | 0.31* | 0.45* | 0.09* | 0.10* | 0.27* |
| Sex (M=1) | -0.08* | 1 | 0.18* | 0.06* | 0.01 | 0.01 | -0.03 | -0.04* |
| BMI | 0.03 | 0.18* | 1 | 0.09* | 0.16* | 0.002 | -0.02 | -0.10* |
| DM | 0.31* | 0.06* | 0.09* | 1 | 0.32* | 0.11* | 0.02 | 0.10* |
| HTN | 0.45* | 0.01 | 0.16* | 0.32* | 1 | 0.13* | 0.03 | 0.11* |
| CKD | 0.09* | 0.01 | 0.002 | 0.11* | 0.13* | 1 | 0.01 | 0.06* |
| Cancer | 0.10* | -0.03 | -0.02 | 0.02 | 0.03 | 0.01 | 1 | -0.003 |
| Dementia | 0.27* | -0.04* | -0.10* | 0.10* | 0.11* | 0.06* | -0.003 | 1 |

*P < 0.01

**2. Why did the authors decide to categorize rather than use continuous variables (i.e. BMI, age as continuous variables)? Such multivariable analyses using continuous variables would add to the literature which typically categorizes BMI.**

**Authors’ response:** Unfortunately, we didn’t have BMI as continuous value. The Korea Disease Control and Prevention Agency (KDCA) provided BMI values as categorized form and did not give us the actual values. It may be due to concerns about personal information. We added a sentence at discussion as limitation.

**Discussion, page 18**

Furthermore, we could not check the distribution of continuous BMI according to fatal illness status. The KCDA provided BMI data in a categorized form.

**3. The authors report missing data in the Results (Table 1) but don’t explain how they handle missing data in the methods. Please clarify there.**

**Authors’ response:** As reported in the table 1, we had missing data about systolic blood pressure (missing n=5), chronic heart disease (16) and dementia (314), respectively. We did not replace the missing numbers so they did not enter multivariable analysis. To clarify this, we added a sentence in the methods and footnotes on the number of patients in the relevant table.

**Methods, Statistical analysis, page 6**

Since dementia had missing data (n = 314) that were not replaced, 3827 participants were included in the multivariable analysis.

**4. Typically, the internal reference group for a cohort study should be the group with the least exposure (i.e. normal weight). While there is sufficient sample size to use the overweight as the reference, I am worried that using overweight instead of normal weight as the internal reference group makes the results confusing to interpret both within the study and in external comparison to other studies that use normal weight as reference. The OR for critical and fatal illness are both non-significant may be interpreted as a higher risk for critical and fatal illness unless very clearly discussed in the abstract, results and discussion. This could paint an impression that overweight is protective, when this may not be the case. For these two reasons, I’d suggest changing the reference group to normal weight. The authors provide some discussion on overweight BMI as a healthy in older adults. However, they do not do any further analysis to test this hypothesis. If they want to keep overweight as the reference category, an analysis of BMI - COVID19 severity/fatality by age (i.e. effect modification by age), would be helpful to determine if there is indeed this age effect.
Authors’ response:** Thank you for the good comment. Regarding the opinion of the reviewer, we conducted statistical analysis based on the Asian-pacific cutoff value and the global standard (Table B). The results still showed U-shaped relationship. The authors discussed a lot about this issue. The overweight group (23.0-24.9 kg/m^2^) of this study is according to the Asian-pacific cutoff value redefined in 2000 and which corresponds to the normal weight according to the global WHO standard. There is still a controversy over the Asian-pacific cutoff value. In 2004, WHO expert consultation recommended that the use of global standards is appropriate because the BMI standards for obesity do not differ greatly by race, and it is not appropriate to stipulate different standards for the Asia-Pacific Region due to small differences. Therefore, it is the opinion of the authors not to change the reference group. However, in order to avoid confusion of global readers, the name of groups such as normal or overweight have been deleted and displayed as numerical values.

Further, the point of this study is to look at the criticality or fatality of covid-19 according to BMI, and it is not to verify the definition of the BMI cutoff value of the Korean or Asian population by comparing it with the WHO standard. However, this is considered to be a very important issue, and it is worth discussing, and the content was added at the discussion as below.

**Discussion, page 13**

Obesity, defined using BMI, has different cutoff values between the global and Asia-Pacific standards. The WHO announced the Asia-Pacific perspective: redefining obesity and its treatment’ in 2000 on the evidence that the Asian population has an increased risk of diseases at BMI > 23 kg/m^2^ [21]. The Asia-Pacific BMI was categorized as followings; underweight (< 18.5 kg/m^2^), normal-weight (18.5-22.9 kg/m^2^), overweight (23.0-24.9 kg/m^2^), obesity-1 (25.0-29.9 kg/m^2^), and obesity-2 (≥ 30 kg/m^2^). Proposed mechanisms of this inter-population discrepancy of defined obesity by BMI include differences in percentage and distribution of body fat across populations, the interplay of genetic susceptibility, and environmental factors related to diet and sedentary lifestyle [19, 20].

However, the debate on whether racial differences exist in defining obesity based on BMI is still ongoing. Despite the differences in BMI cutoff points for obesity in these populations, a meta-analysis of four continents in 239 prospective studies showed that BMI and all-cause mortality were broadly consistent across the four continents [3]. In 2004, WHO expert consultation recommended that the use of global standards is appropriate because the BMI standards for obesity do not differ greatly by race, and it is not appropriate to stipulate different standards for the Asia-Pacific region due to small differences [19]. In a study comparing the BMI cutoff value of the South Koreans with the global standard in 2015, the cutoff point of Korean BMI was 24.2 kg/m^2^ (sensitivity 78%, specificity 71%, as the result of receiver operating characteristic cure, based on the body fat percentage) and it was only 1.3 kg/m^2^ lower than the global standard [25]. The result supports the recommendation of the WHO expert consultation in 2004. Nonetheless, the Korean government continues to follow the 2000 Asian-Pacific cut-off values. Hence, to avoid confusion, we did not express obesity or normal for the BMI group, but only used numeric values in this study.

**Table B. Multivariable regression according to the different BMI reference**

|  | **Critical illness (original data)** | **Critical illness (Asian)** | **Critical illness (Global)** |
| --- | --- | --- | --- |
|  | **Odds ratio (95% CI)** | **Odds ratio (95% CI)** | **Odds ratio (95% CI)** |
| **Age** | 2.64 (2.20-3.15) | 2.64 (2.20-3.15) | 2.64 (2.21-3.16) |
| **Male (vs. female)** | 2.85 (1.94-4.19) | 2.85 (1.94-4.19) | 2.79 (1.90-4.09) |
| **BMI (kg/m^2^)** |  |  |  |
| <18.5 | 3.29 (1.54-7.04) | 2.07 (1.05-4.09) | 2.41 (1.24-4.68) |
| 18.5-22.9 | 1.59 (0.94-2.71) | 1 (Reference) | 1 (Reference) |
| 23-24.9 | 1 (Reference) | 0.63 (0.37-1.07) |  |
| 25.0-29.9 | 2.37 (1.38-4.07) | 1.49 (0.94-2.36) | 1.79 (1.81-2.71) |
| ≥30 | 4.40 (1.66-11.66) | 2.76 (1.09-7.00) | 3.31 (1.33-8.22) |
| **Diabetes mellitus** | 1.95 (1.32-2.86) | 1.95 (1.32-2.86) | 1.90 (1.30-2.80) |
| **Hypertension** | 1.64 (1.11-2.43) | 1.64 (1.11-2.43) | 1.61 (1.09-2.38) |
| **Chronic kidney disease** | 3.12 (1.36-7.19) | 3.12 (1.36-7.19) | 3.17 (1.38-7.82) |
| **Cancer** | 2.28 (1.12-4.62) | 2.28 (1.12-4.62) | 3.17 (1.38-7.28) |
| **Dementia** | 2.13 (1.25-3.63) | 2.13 (1.25-3.63) | 2.22 (1.30-3.78) |

**Results general comments/questions:
1. At present the tables and figures do not provide sufficient information/footnotes to stand on their own for interpretation. For instance, in Table 2, the footnote needs to indicate that age is for every 10 years (categorical instead of continuous) and provide details on how critical and fatal illness are defined, how co-morbid conditions are defined, and what covariates were used in analysis**

**Authors’ response:** As you recommend, we added a footnote to table 2 and figures. Detailed information regarding critical and fatal illness were describe at the section of ‘study definition and outcome assessment’ in Method, page 5. As we already responded previous question, the co-morbid condition was collected through history taking by medical personnel. There was no detailed information and it might be previously diagnosed at a medical institution. We described about multivariable analysis including lists of five covariates in ‘statistical analysis’ section, page 6.

**Results, table 2 footnote, page 10**

*n= 3827, The ORs of multivariable analyses were adjusted for age, sex, and five comorbidities (diabetes mellitus, hypertension, chronic kidney disease, cancer, and dementia). **Age was given as a categorical variable in units of 10 years, but we calculated the odds ratio of every 10 years as a continuous variable. BMI, body mass index; CKD, chronic kidney disease

**Results, Figure 1 &3 footnote, page 11-12**

**Figure 1. Odds ratios for critical and fatal illness according to body mass index. Odds ratios were adjusted for age, gender, and five comorbidities (diabetes mellitus, hypertension, chronic kidney disease, cancer, and dementia).**

**Figure 2. Odds ratios for critical and fatal illness of male and female according to body mass index (A) critical illness, (B) fatal illness Odds ratios were adjusted for age and five comorbidities (diabetes mellitus, hypertension, chronic kidney disease, cancer, and dementia).**

**Figure 3. Subgroup analysis for fatal illness according to the BMI in patients with/without diabetes mellitus or hypertension (A) Diabetes Mellitus*, (B) Hypertension**. *Odds ratios were adjusted for age, gender, and four comorbidities (hypertension, chronic kidney disease, cancer, and dementia). **Odds ratios were adjusted for age, gender, and four comorbidities (diabetes mellitus, chronic kidney disease, cancer, and dementia).**

**2. Figure 2: It is surprising to me that these two have the exact same p-interaction value (for female/male and critical illness, fatal illness) to the 0.0001 confidence level. I’d advise the authors double check this and confirm, especially considering how different the trends/figures look.
Authors’ response:** Thank you for good advice and sorry for the mistake. The two graphs in Figure 2 were almost identical, only differences due to change in the reference group. Our statistician recommends that this type of figure presentation is unusual and uninformative, so we modified it to:

**Figure 2. Odds ratios for critical and fatal illness of male and female according to body mass index**

**(**A) Odds ratios for critical illness, (B) Odds ratios for fatal illness

**3. Include 95% CI in the narrative text in addition to the tables and figures
4. Improve interpretation sentences for odds ratios.**

**Authors’ response:** As you recommend, we added 95% CI in the narrative text.

**Result, page 9**

With every 10 years of increase in age, the odds ratio of multivariable analyses for critical and fatal illness increased to 2.64 (95% CI: 2.20-3.15) and 3.04 (2.45-3.78), respectively. Male sex was also found to be an independent risk factor. When the BMI of 23.0-24.9 kg/m^2^ group was used as a reference, the risk of BMI ranging 18.5-22.9 kg/m^2^ was as high as 1.59 (0.88-2.89) for fatal illness without statistical significance.

**Discussion general comments/questions:
1. The authors include new results from the sub-group analyses in their discussion instead of summarizing and interpreting findings from their results.**

**Authors’ response:** As you recommend, we deleted S2-4 figures and made some changes for the sub-group analyses. We performed subgroup analyses regarding whether they have DM/HTN or not and repositioned the data to the result section.

**Result, page 11-12**

Similar non-linear relationships are shown in Fig 3. This figure shows subgroup analyses of subjects with (A) DM or (B) hypertension. Each group showed a non-linear pattern with increasing BMI. In addition, there was no significant difference according to the presence or absence of the diseases (*P*_interaction_ = 0.62 for DM_,_ *P*_interaction_ = 0.19 for hypertension)

**Figure 3. Subgroup analysis for fatal illness according to the BMI in patients with/without diabetes mellitus or hypertension** (A) Diabetes Mellitus*, (B) Hypertension**. *****Odds ratios were adjusted for age, gender, and four comorbidities (hypertension, chronic kidney disease, cancer, and dementia). ******Odds ratios were adjusted for age, gender, and four comorbidities (diabetes mellitus, chronic kidney disease, cancer, and dementia).

**2. The authors focus their results on the literature around BMI and all-cause mortality. This is somewhat related, but it would bet better to review literature more specific to BMI and infection-related fatality.**

**Authors’ response:** As you recommend, we had more specified the discussion.

**Discussion, page 14**

There are limited studies on the clinical implications of BMI, from underweight to obesity, in viral pandemics. In the case of influenza-associated pneumonia, an increase in risk has been observed in underweight and obesity [4]. However, regarding obesity, contradictory results–obesity paradox-were reported in previous studies [15]. Similarly, there was a recent study on ‘an obesity survival paradox’ that authors showed the projected rates of COVID-19 infection and mortality drop with elevated prevalence of obesity in United States [26]. However, in the current COVID-19 pandemic, the majority of studies reported obesity as a risk factor for progressing to severe COVID-19 and were associated with the need for hospitalization and admission to the critical care unite due to COVID-19 [7, 17, 27, 28]. Importantly, our study also showed increased BMI as a risk factor for critical and fatal illness of COVID-19 and cannot prove the ‘obesity survival paradox.’

In contrast to obesity, the relationship between underweight and COVID-19 is unknown. A recent study showed a J-shaped (non-linear) relationship between BMI and risk for COVID-19 related hospitalization, ICU admission, and death [28]. In our study, subjects with a BMI ≥ 30 kg/m^2^ presented higher fatality with an odds ratio of 4.32, compared to subjects of a 23 < BMI < 25 kg/m^2^. In addition, BMI < 18.5 kg/m^2^ also showed an odds ratio of 3.97 (1.77-8.92) for fatal illness.

**3. No mention of alternative exposure measures for overweight/obesity like waist circumference or waist to hip ratio. Even if not possible with this data set, it would be good to mention limitations of BMI as an exposure measure.**

**Authors’ response:** We added some discussion with new references.

**Discussion, page 14-15**

One recent remarkable study is the analysis of the UK biobank, even though preliminary data are available [29, 30]. They showed that BMI was strongly associated with positive results in the COVID-19 test and the risk of death related to COVID-19 [29]. Interestingly, both BMI and waist circumference were associated with testing positive for COVID-19 in a dose-response fashion [30]. However, another study using a two-sample multivariable Mendelian randomization failed to show the impact of body composition (waist circumference, trunk fat ratio) on COVID-19 susceptibility and severity; only BMI was significant [31]. Our study has a limitation in that it did not include body composition.

**4. Need more discussion on small sample size for underweight and obese II and how miss affected the certainty in the odds ratio estimates.
Authors’ response:** We added this at Discussion as limitation.

**Discussion, page 18**

Furthermore, detailed information about comorbidities was not provided. The number of patients with BMI < 18.5, or ≥ 30 kg/m^2^, was small; 191 (4.6%) or 193 (4.7%), respectively. Therefore, there is a chance of being statistically underpowered or having an increased margin of error. Furthermore, we could not check the distribution of continuous BMI according to fatal illness status. The KCDA provided BMI data in a categorized form.

**I suggest the authors add into their discussion and intro this new U.S. BMI and COVID-19 severity and death, which found a similar non-linear relationship between BMI and COVID-19 severity/death (increased risk at underweight and obese level BMIs)**

**Authors’ response:** Thank you for good information. We added some discussion with a new reference.

**Discussion, page 14**

In contrast to obesity, the relationship between underweight and COVID-19 is unknown. One recent paper showed a J-shaped (non-linear) relationship between the BMI and risk for COVID-19 related hospitalization, ICU admission and death [26]. In this study, obesity-2 (BMI ≥ 30 kg/m^2^) presented higher fatality odds ratio of 4.32 compared to overweight

Line specific Comments:=============================================
**Abstract:
32- 34: No mention of OR for normal weight in the abstract.
36-37: Conclusion explains underweight and obese have increased risk for critical/fatal illness than normal and overweight, but OR is only for overweight as reference.**

**Authors’ response:** We made a change as follows;

**Abstract, page 2**

Results: Among participants, those with a BMI of 18.5-22.9 kg/m^2^ were the most common (42.0%), followed by 25.0-29.9 kg/m^2^ (24.4%), 23.0-24.9 kg/m^2^ (24.3%), ≥ 30 kg/m^2^ (4.7%), and < 18.5 kg/m^2^ (4.6%). In addition, 1,654 (41.2%) were men and 3.04% were fatalities. Multivariable analysis showed that age, male sex, BMI < 18.5 kg/m^2^, BMI ≥ 25 kg/m^2^, diabetes mellitus, chronic kidney disease, cancer, and dementia were independent risk factors for fatal illness. In particular, BMI < 18.5 kg/m^2^ (odds ratio [OR] 3.97, 95% CI 1.77-8.92), 25.0-29.9 kg/m^2^ (2.43, 1.32-4.47), and ≥ 30 kg/m^2^ (4.32, 1.37-13.61) were found to have higher ORs than the BMI of 23.0-24.9 kg/m^2^ (reference). There was no significant difference between those with a BMI of 18.5-22.9 kg/m^2^ (1.59, 0.88-2.89) and 23.0-24.9 kg/m^2^.

Conclusions: This study demonstrated a non-linear (U-shaped) relationship between BMI and fatal illness. Subjects with a BMI of < 18.5 kg/m^2^ and those with a BMI ≥ 25 kg/m^2^ had a high risk of fatal illness. Maintaining a healthy weight is important not only to prevent chronic cardiometabolic diseases, but also to improve the outcome of COVID-19.

**Introduction:
45 : poor grammar – “as the increasing period of social distancing”, “more like to eat unhealthy food”**

**Authors’ response:** We took English editing and made a change like this ‘Nowadays, people are more likely to gain weight as a result of increasing social distancing, increasing intake of unhealthy food, and decreased physical activity [1].

**48-50: Its unclear why the author is mentioning U-shaped curve for BMI and all cause, CVD mortality, when the outcome here is COVID-19 related severe illness and fatality. Better to remove and cite literature on the association between BMI and flu-like virus severe illness and fatality (which you do a bit later).**

**Authors’ response:** You can check the issue following paragraph

**51-57: There is now far more global data that can be cited regarding cohorts of COVID-19 severity and hospitalization with BMI as a risk factor. Consider citing others (particularly within Asia region for comparability)**

**Authors’ response:** We already cited China study and added more valuable references. However, there was little available data within Asia region regarding this topic.

**58: clarify if cardiac disease means Cardiovascular disease (CVD)?**

**Authors’ response**: The study is a systemic review and meta-analysis. The authors just used the term of ‘cardiac diseases’ for cardiovascular disease/coronary artery disease. Therefore, it’s better to use the same terms used by the original authors.

**65-66 : bad English grammar
68: “associated with COVID-19…” and “obesity as a risk factor for COVID-19” Be more specific. Are you referring to COVID-19 infection or COVID-19 severe illness/fatality? I don’t know of any evidence suggesting obese have a higher risk for being INFECTED, though there is substantial data for SEVERE ILLNESS/FATALITY?**

**Authors’ response:** We a change like this ‘To date, there is scarcity of information regarding the obesity survival paradox or underweight risk associated with the outcomes of COVID-19.’

**74-75: this line about Korean vs. Westerns having lower obesity and higher underweight prevalence belongs earlier in the introduction. Move and add more about the prevalence (cite national level stats for Korea)**

**Authors’ response:** This is not the important issue for study introduction. We deleted the sentence.

**Data Source and Study Population
83: “almost all confirmed cases” – what does this mean? Can you provide an estimate of the percentage of tracking currently occurring?**

**Authors’ response:** We have detailed number of subjects and a Figure about the total number of COVID-19 tests (623,069) and confirmed cases (9,320) at the time of current study. Among the confirmed patients who initially admitted to a community treatment center and transferred to the hospital, **about 10 subjects was unclear.** However, the data and the figure were under review so we cannot show the figure.

**88-91: how has the exclusion of 1487 patients affected representativeness of the data? In particular, are those individuals without available BMI values systematically different than those with BMI data? This could lead to serious selection bias. Include tables/data on representativeness of selected data vs. missing data.**

**Authors’ response:** As your advice, we compared the selected data and missing data.

In Table C, we compared the subjects with and without BMI value and calculated the odds ratios for BMI missing using uni/multivariable logistic regression (Table C).

**Table C. Demographic data of with/without BMI value and multivariable analysis for comparing the data with/without BMI value**

|  | With BMI | Without BMI | Univariable | | Multivariable* | |
| --- | --- | --- | --- | --- | --- | --- |
|  | N=4141, (%) | N=1166, (%) | OR** | 95% CI | OR** | 95% CI |
| age (y)* |  |  |  |  |  |  |
| 20-29 | 942 (22.7) | 162 (13.9) | 1 (ref) |  | 1 (ref) |  |
| 30-39 | 469 (11.3) | 80 (6.9) | 1.99 | 0.74-1.33 | 0.94 | 0.70-1.27 |
| 40-49 | 595 (14.4) | 143 (12.3) | 1.40 | 1.09-1.79 | **1.33** | **1.03-1.71** |
| 50-59 | 873 (21.1) | 268 (23.0) | 1.79 | 1.44-2.22 | **1.66** | **1.33-2.08** |
| 60-69 | 674 (16.3) | 232 (19.9) | 2.00 | 1.60-2.50 | **1.79** | **1.41-2.28** |
| 70-79 | 378 (9.1) | 167 (14.3) | 2.57 | 2.01-3.29 | **2.03** | **1.54-2.68** |
| ≥80 | 210 (5.1) | 114 (9.8) | 3.16 | 2.38-4.19 | **1.89** | **1.32-2.71** |
| Female | 2415 (58.3) | 729 (62.5) | 0.84 | 0.73-0.96 | 0.88 | 0.77-1.01 |
| DM | 501 (12.1) | 183 (15.7) | 1.35 | 1.13-1.63 | 0.96 | 0.78-1.17 |
| HTN | 862 (20.8) | 335 (28.7) | 1.53 | 1.32-1.78 | 1.07 | 0.90-1.28 |
| HF | 40 (1.0) | 19 (1.6) | 0.70 | 0.98-2.95 | 1.11 | 0.63-1.97 |
| CCD | 134 (3.2) | 45 (3.9) | 1.20 | 0.85-1.69 | 0.88 | 0.62-1.26 |
| asthma | 98 (2.4) | 28 (2.4) | 1.02 | 0.66-1.55 | 0.92 | 0.59-1.42 |
| COPD | 29 (0.7) | 9 (0.8) | 1.10 | 0.52-2.34 | 0.76 | 0.35-1.65 |
| CKD | 44 (1.1) | 11 (0.9) | 0.89 | 0.46-1.72 | 0.63 | 0.31-1.25 |
| cancer | 107 (2.6) | 38 (3.3) | 1.27 | 0.87-1.85 | 1.01 | 0.69-1.48 |
| dementia | 121 (2.9) | 103 (8.8) | 2.97 | 2.26-3.90 | **2.26** | **1.36-3.13** |

*adjusted with age, sex, DM, HTN, HF, CCD, asthma, COPD, CKD, cancer, dementia

**OR for BMI missing

The result showed high rate of old age and dementia (which was independent risk factor for fatal illness) in BMI missing group. The BMI missing group showed higher fatal illness (9.9% vs. 3.0%) which are probably due to age and dementia differences. Although, this is not the well-designed prospective study and has some limitations. We don’t think this difference is a severe selection bias that is enough to reverse our results.

We randomly assigned 234 subjects from the BMI missing group to each of the 5 BMI groups and performed a multivariable analysis, and the pattern of the results (U-shaped relationship) did not change. Further, we assigned all BMI missing subjects (1164) in each BMI group and still showed nonlinear relationship and only when the BMI missing subjects totally assigned in reference BMI group (23.0-24.9kg/m^2^) lost statistical significance.

**Table D; multivariable analysis for fatal illness (adjusted age, sex, five comorbidities)**

1. Randomly assigned 234 subjects from BMI missing group to each BMI group of original data

2. Assigned all subjects of BMI missing (1164) into BMI < 18.5 group

3. Assigned all subjects of BMI missing (1164) into BMI 18.5-22.9 group

4. Assigned all subjects of BMI missing (1164) into BMI 23.0-24.9 group

5. Assigned all subjects of BMI missing (1164) into BMI 25.0-29.9 group

6. Assigned all subjects of BMI missing (1164) into BMI ≥ 30 group

|  | **1** | **2** | **3** | **4** | **5** | **6** |
| --- | --- | --- | --- | --- | --- | --- |
|  | **OR (95% CI)** | **OR (95% CI)** | **OR (95% CI)** | **OR (95% CI)** | **OR (95% CI)** | **OR (95% CI)** |
| **Age** | 3.1 (2.6-3.6) | 3.0 (2.6-3.5) | 3.0 (2.5-3.5) | 3.0 (2.6-3.5) | 3.1 (2.6-3.6) | 3.0 (2.6-3.6) |
| **Male (vs. female)** | 2.5 (1.9-3.5) | 2.7 (2.0-3.7) | 2.7 (1.9-3.6) | 2.5 (1.8-3.4) | 2.6 (1.9-3.5) | 2.7 (2.0-3.7) |
| **BMI (kg/m^2^)** |  |  |  |  |  |  |
| <18.5 | 2.8 (1.7-4.8) | 4.5 (2.6-7.6) | 3.9 (1.8-8.5) | 1.2 (0.6-2.4) | 3.7 (1.7-8.1) | 3.8 (1.7-4.4) |
| 18.5-22.9 | 1.3 (0.8-2.0) | 1.6 (0.9-2.8) | 3.0 (1.8-5.0) | 0.5 (0.4-0.8) | 1.5 (0.9-2.8) | 1.6 (0.9-2.8) |
| 23-24.9 | 1 (Reference) | 1 (Reference) | 1 (Reference) | 1 (Reference) | 1 (Reference) | 1 (Reference) |
| 25.0-29.9 | 2.2 (1.4-3.4) | 2.4 (1.3-4.4) | 2.4 (1.3-4.4) | 0.8 (0.5-1.3) | 3.7 (2.2-6.2) | 2.4 (1.3-4.4) |
| ≥30 | 2.4 (1.3-4.3) | 4.1 (1.3-12.7) | 4.1 (1.4-12.6) | 1.4 (0.5-3.9) | 4.2 (1.4-12.9) | 4.6 (2.7-7.8) |
| **DM** | 2.1 (1.5-2.9) | 2.2 (1.6-3.0) | 2.2 (1.6-3.0) | 2.1 (1.5-2.8) | 2.1 (1.5-2.9) | 2.2 (1.6-3.0) |
| **Hypertension** | 1.3 (0.9-1.7) | 1.3 (0.9-1.7) | 1.3 (0.9-1.8) | 1.3 (0.9-1.7) | 1.2(0.9-1.7) | 1.2 (0.9-1.7) |
| **Chronic kidney disease** | 2.4 (1.1-5.0) | 2.5 (1.2-5.6) | 2.3 (1.1-4.7) | 2.4 (1.1-5.1) | 2.4 (1.1-5.2) | 2.5 (1.2-5.0) |
| **Cancer** | 2.8 (1.6-5.0) | 2.9 (1.6-5.1) | 2.7 (1.5-4.7) | 3.0 (1.7-5.3) | 2.9 (1.6-5.2) | 2.9 (1.6-5.1) |
| **Dementia** | 2.4 (1.6-3.5) | 2.2 (1.5-3.2) | 2.3 (1.6-3.4) | 2.5 (1.7-3.7) | 2.4 (1.6-3.5) | 2.2 (1.5-3.3) |

**Statistical Analysis
118: incorrect use of the term multivariate- this is a multivariable** logistic regression. Multivariate refresh to modeling of data that are often derived from longitudinal studies (repeated measures) which this is not.**

**118: should be COVID-19 critical illness and COVID-19 fatal illness**

**Authors’ response:** As your advice, we corrected the errors.

**127- 131: language around the stratified analyses is unclear at present. It sounds like the authors did an overall multivariable logistic regression, controlling for all potential confounders and then did stratified analyses by sex and each co-morbid condition separately. Add in language around stratified analysis for greater clarity.**

**129-131: this last statement is also confusing. It is unclear from what the authors are saying whether, when stratifying by each comorbidity, they are calculating the odds ratios only for patients who do vs. do NOT have the target disease?**

**Authors’ response:** As you understood, we did an overall multivariable logistic regression, controlling for all potential confounders and then did stratified analyses by sex and each co-morbid condition separately. Due to the limited number of specific comorbidities and also to rule out the specific disease effect, odds ratios of patients who did not have the target disease were calculated.

Sorry for the confusion, we deleted S2-4 fig which present subgroup analysis and newly added fig 3 which presents subgroups with/without DM or with/without HTN according to the advice of a statistician.

We changed the sentences like this ‘To analyze the relationship between BMI and fatality by sex, we performed a multivariable analysis after adjusting for age and the five comorbid conditions. In addition to sex, we analyzed the relationship between BMI and fatality according to whether they had DM or HTN. We performed multivariable analysis after adjusting for age, sex, and four comorbidities other than the target disease (DM or HTN). Then, the relationships between BMI and fatality in the groups with and without the diseases (DM or HTN) were evaluated separately.’

**Table 1
- Include percentage of total population for each BMI (i.e. 1741 is 42% of total patients) as you have written this in your narrative results.**

**Authors’ response:** As your advice, we added the percentage of each BMI of Table 1.

**Is the p-value for the statistically significant differences within a given covariate (age group, sex, etc.) across BMI categories? Or is it a p-value for statistically significant differences within a BMI category across covariates (across age groups, sexes, etc.). For the results text it seems to be the former, but please clarify in methods and in table footnotes what the p-value refers to**

**Authors’ response:** It was descriptive statistics which was mentioned in statistical analysis. It has slightly changed the format of Table 1 for better readability.

**161: start new paragraph when start talking about the association/odds ratio results. “Fatal illness was significantly related to the combined comorbidity” – unclear what this means as written.**

**Authors’ response:** We deleted the sentence and made a new paragraph.

**175 -176: unclear what the authors mean by “did not change the aspect of underweight”. This is not common statistical language.**

**Authors’ response:** This sentence was to compare fig 1 and to S1 fig. We change the sentence for better readability. 🡪 After multivariable analysis with adjusting covariates, a similar result was shown in the group with BMI < 18.5 kg/m^2^ (Fig 1).

**Figure 2: It is surprising to me that these two have the exact same p-interaction value (for female/male and critical illness, fatal illness) to the 0.0001 confidence level. I’d advise the authors double check this and confirm, especially considering how different the trends/figures look.**

**Authors’ response:** The same question which was already answered.

**Discussion:
203-209: This review of sub-group analyses should be moved to the results section. The authors should only include a short summary of this analysis and its implications in the discussion section.**

**Authors’ response:** As already answered, we deleted S2-4 figures and made some changes for the sub-group analyses. We performed subgroup analyses regarding whether they have DM or HTN and repositioned the data to the result section.

**223-224: It is not clear what the authors mean by “contradictory results were reported in obesity according to other studies”. I suggest they make this clear.**

**225-244: Remove or reduce the detailed comparison to UK literature on BMI and overall mortality (non-specific to infection). A mention of the overall association between BMI and mortality is ok, but not a detail comparison as the authors here are specifically looking at COVID-19 severe illness and fatality. Better to compare to literature on similar viral infections or COVID19 studies.
245-255: same is true here. The discussion should be focused more on literature about the actual exposures and outcomes of interest in this paper, not a more general topic like all cause mortality.**

**Authors’ response:** As your advice, we removed the sentence and focused on the topic. “Contradictory results” means obesity paradox, we clarified this adding some sentences and references.

**256: “The BMI value for healthy weight varied with age” – is this a finding of the analysis or something gleaned from the literature. As written, it is confusing whether this is a general statement or a specific finding.**
**258-259: This is an interesting statement. Did the authors do any analysis to confirm whether there was effect modification by age for the OR in overweight vs. normal weight in the study. Would be necessary to confirm this with data.**

**Authors’ response:** We removed the sentence.

**266-268: poor English grammar, consider revising.
266-284: discussion around Asian specific BMI cut points – did the authors consider doing a sensitivity analysis with the general WHO BMI cut-points? This would be a helpful comparison to see how much these differential BMI cut-points made a difference in the reported odds ratios.
279: typo – should be 25.0-29.9 kg/m2, not 2.5-29.9 kg/m2**

**Authors’ response:** We removed the whole paragraph and made a change according to the previous question.

**286-287: mention of how obesity potentiates multiple cardiovascular risk factors, but they did not discuss how they addressed this in their methods. See general statistical analysis question above**

**Authors’ response:** We were already answered.

**315: the authors looked at the relationships between BMI and critical and fatal illness from COVID-19 (not just fatality from COVID-19). Revise how this is written.**

**Authors’ response:** As you mentioned, we also analyzed the relationship between BMI and critical illness. Due to the small number of fatal illnesses, we considered the possibility that the relationship between BMI and fatal illness would be statistically underpowered or increased the margin of error. Therefore, critical illness was defined as a broader term, including fatal illness, and was confirmed to see if the relationship between a larger number of critical illnesses and BMI also showed a similar pattern to fatal illness. Anyway, you changed our title.
